# Maternal oral probiotic use is associated with decreased breastmilk inflammatory markers, infant fecal microbiome variation, and altered recognition memory responses in infants—a pilot observational study

**DOI:** 10.3389/fnut.2024.1456111

**Published:** 2024-09-25

**Authors:** Sara Gonia, Timothy Heisel, Neely Miller, Jacob Haapala, Lisa Harnack, Michael K. Georgieff, David A. Fields, Dan Knights, Katherine Jacobs, Elisabeth Seburg, Ellen W. Demerath, Cheryl A. Gale, Marie H. Swanson

**Affiliations:** ^1^Department of Pediatrics, University of Minnesota, Minneapolis, MN, United States; ^2^Department of Psychology, University of Minnesota, Minneapolis, MN, United States; ^3^Division of Epidemiology and Community Health, School of Public Health, University of Minnesota, Minneapolis, MN, United States; ^4^Harold Hamm Diabetes Center, University of Oklahoma Health Sciences Center, Oklahoma City, OK, United States; ^5^Department of Computer Science and Engineering, University of Minnesota, Minneapolis, MN, United States; ^6^Division of Maternal-Fetal Medicine, University of Minnesota, Minneapolis, MN, United States; ^7^Pregnancy and Child Health Research Center, HealthPartners Institute, Bloomington, MN, United States

**Keywords:** probiotics, gut microbiome, Infant, Inflammation, neurodevelopment, event related potential, breastmilk

## Abstract

**Introduction:**

Early life gut microbiomes are important for brain and immune system development in animal models. Probiotic use has been proposed as a strategy to promote health via modulation of microbiomes. In this observational study, we explore if early life exposure to probiotics via the mother during pregnancy and lactation, is associated with decreased inflammation in breastmilk, maternal and infant microbiome variation, and altered infant neurodevelopmental features.

**Methods:**

Exclusively breastfeeding mother-infant dyads were recruited as part of the “Mothers and Infants Linked for Healthy Growth (MILk) Study.” Probiotic comparison groups were defined by exposure to maternal probiotics (NO/YES) and by timing of probiotic exposure (prenatal, postnatal, total). C-reactive protein (CRP) and IL-6 levels were determined in breastmilk by immunoassays, and microbiomes were characterized from 1-month milk and from 1- and 6-month infant feces by 16S rDNA sequencing. Infant brain function was profiled via electroencephalogram (EEG); we assessed recognition memory using event-related potential (ERP) responses to familiar and novel auditory (1 month) and visual (6 months) stimuli. Statistical comparisons of study outcomes between probiotic groups were performed using permutational analysis of variance (PERMANOVA) (microbiome) and linear models (all other study outcomes), including relevant covariables as indicated.

**Results:**

We observed associations between probiotic exposure and lower breastmilk CRP and IL-6 levels, and infant gut microbiome variation at 1- and 6-months of age (including higher abundances of *Bifidobacteria* and *Lactobacillus*). In addition, maternal probiotic exposure was associated with differences in infant ERP features at 6-months of age. Specifically, infants who were exposed to postnatal maternal probiotics (between the 1- and 6-month study visits) via breastfeeding/breastmilk, had larger differential responses between familiar and novel visual stimuli with respect to the late slow wave component of the EEG, which may indicate greater memory updating potential. The milk of mothers of this subgroup of infants had lower IL-6 levels and infants had different 6-month fecal microbiomes as compared to those in the “NO” maternal probiotics group.

**Discussion:**

These results support continued research into “Microbiota-Gut-Brain” connections during early life and the role of pre- and postnatal probiotics in mothers to promote healthy microbiome-associated outcomes in infants.

## 1 Introduction

The early life gut microbiome co-develops in concert with aspects of host physiology such as mucosal barrier function, metabolism, immunity and brain function. The significance of this co-development is highlighted by studies in animal models that have shown that disruptions in early-life microbiomes are mechanistically linked to abnormal development of these physiological systems (1–4). In humans, infant gut microbiome composition is affected by maternal microbiomes (via transfer of microbes), birth mode, infant diet and environmental exposures (5–8). Based on the importance of microbiomes in health, there has been increasing research interest in designing strategies to promote healthy microbiomes during infancy, a sensitive period for brain development. The proposed goal of early microbiome modulation is, ideally, to improve both short and long-term health outcomes, particularly in infants that are at risk for microbiome disruption. Consistent with the Developmental Origins of Health and Disease (DOHaD) hypothesis, modulation strategies that can be targeted to the birth parent at very early times in development (e.g., prior to or during pregnancy) offer the possibility for greatest offspring health benefit (9–12).

One therapeutic strategy that has been proposed to promote healthy early life microbiomes is probiotic administration to women during pregnancy and lactation. Probiotic supplementation during pregnancy has been demonstrated to be safe for both mother and infant (13, 14), with several studies showing that probiotic use during pregnancy and lactation modulates breastmilk and/or infant gut microbiota (15–17). In pregnant women, prenatal probiotics are associated with improved metabolism and pregnancy outcomes including reduced incidences of gestational diabetes, preeclampsia, vaginal infections, and preterm labor (18–21). Prenatal probiotics have also been associated with health benefits in offspring, specifically with respect to gut health, the development of immunity, and reduced atopic disease (22–26). An association has been shown between a mother’s intestinal microbiota and their serum inflammation biomarkers, both of which may contribute to newborn early life immune and metabolic programming, independent from probiotic exposure, highlighting a potential broader role for maternal microbes and inflammation to affect offspring health (27). Connections between probiotic use during pregnancy/lactation and host and offspring inflammatory markers is an emerging theme of current research (28–30).

Research in animal models supports a role for gut microbes in modulating brain function (i.e., the Microbiome-Gut-Brain Axis) across the lifespan (31–33). Germ-free mice, as well as mice with perturbed gut microbiomes due to antibiotic exposure, exhibit deficits in social functioning and memory (34, 35) and altered anxiety and motor responses (1). Importantly, developing (young) brains have been shown to be far more susceptible to the effects of microbiome modulation than mature brains (1), reinforcing the important concept that there is an early-life sensitive period for brain developmental programming by the microbiome. The hippocampus, a brain structure critical to recognition memory and cognitive function, is the target of many of these gut microbial-mediated effects (1, 35, 36). For example, adult germ-free mice exhibit defects in memory (35), altered hippocampus levels of the neurotransmitter serotonin (36), and altered expression of hippocampus gene transcripts (1). Dietary supplementation with specific bacteria (e.g., *Lactobacillus* and *Bifidobacteria*) has been shown to enhance memory in adult rats (37). These exciting mechanistic connections among gut microbes, probiotics, and early life brain function in animal models have not yet been translated to human infants.

In human infants, functional maturation of the hippocampus and other medial temporal lobe structures responsible for recognition memory processing is evaluated by recording and analyzing the brain’s electrophysiologic response to familiar vs. novel stimuli using electroencephalogram (EEG), or event-related potential (ERP) responses (38, 39). The ERP waveform is the portion of the EEG that reflects cognitive processing of a stimulus. It is embedded in the raw EEG data and is extracted by averaging the EEG across multiple presentations of the stimulus (38). Studies of infants at risk for neurodevelopmental impairment have demonstrated that recognition memory can be evaluated in very young (newborn) infants and, importantly, that early recognition memory is associated with later recognition and behavioral memory performance (40, 41) and cognitive function (42). Previous research has also shown the predictive value of early ERP for later language development, reading ability, and risk for autism (43–46).

In this pilot observational study, we explore associations between maternal probiotic exposure (and its timing) with infant brain (recognition memory) function as measured by ERPs, specifically the electrophysiological responses recorded at the scalp in response to familiar and novel auditory and visual stimuli. We also compare inflammatory markers in breastmilk as well as milk and infant fecal microbiome features with respect to maternal probiotic exposure to gain insight into potential mechanistic links among inflammation, microbiomes, and brain function.

## 2 Materials and methods

### 2.1 Subject enrollment and inclusion criteria

Mother-infant dyads were enrolled as part of the Mothers and Infants Linked for Healthy Growth (MILk) study, a multi-site study in Oklahoma City, Oklahoma and the Minneapolis/St. Paul metropolitan area in Minnesota as previously described (47). The participants in the present study included a subset of MILk Study dyads who consented to additional microbiome assessments of their milk and their infants’ feces. Briefly, dyads were healthy, with mothers meeting the following inclusion criteria: 21–45 years of age, non-smoking, non-alcohol drinking, non-diabetic, English speaking and understanding, and delivered singleton infants at term gestation (37 0/7–42 1/7 weeks) who were appropriately grown for gestational age (between the 10^th^ and 90^th^ percentile on WHO growth charts). Infants were exclusively breastfed (EBF) through 6 months of age, with EBF defined as being fed only human milk and < 24 oz. (720 mL) of formula or other liquids since birth or their last study visit. None of the women reported symptoms of mastitis or breast infection at the time of breastmilk collection.

### 2.2 Clinical and demographic variable definitions and collection methods

Clinical and demographic data were obtained via the electronic health record from prenatal care and the birth hospitalization as well as from electronic questionnaires administered to mothers at the study visits at 1 and 6 months of infant age and are listed in Supplementary Table 1. Study questionnaires did not ask about parent gender identity and readers of this paper should be encouraged to read/use the gender-associated terms in this paper according to those with which they most identify, per the guidance of the Academy of Breastfeeding Medicine. Maternal pre-pregnancy body mass index (BMI) was defined as healthy (18.5–25), overweight (25–30), or obese (> 30). Normal versus excess gestational weight gain (GWG) was defined as developed by the American College of Obstetrics and Gynecology (American College of Obstetrics and Gynecology, 2013). The healthy eating index (HEI) is a measure of diet quality as developed by the USDA (48–50). Maternal and infant antibiotic exposure as yes/no variables were defined as follows. Maternal prenatal antibiotic exposure was considered “yes” if occurring during pregnancy and up until (not including) the birth hospitalization based on participant report. Maternal and infant postnatal antibiotics were considered “yes” at 1 month of infant age if occurring during the birthing process (including antibiotics administered due to C-section and/or Group B Streptococcus (GBS) positive maternal status) and up to and including the 1-month sample collection time. Maternal and infant postnatal antibiotic exposures were considered “yes” at 6-months of infant age if occurring after the 1-month and up to the 6-month sample collection time.

### 2.3 Development of probiotic groups and timing categories

**Figure 1:**
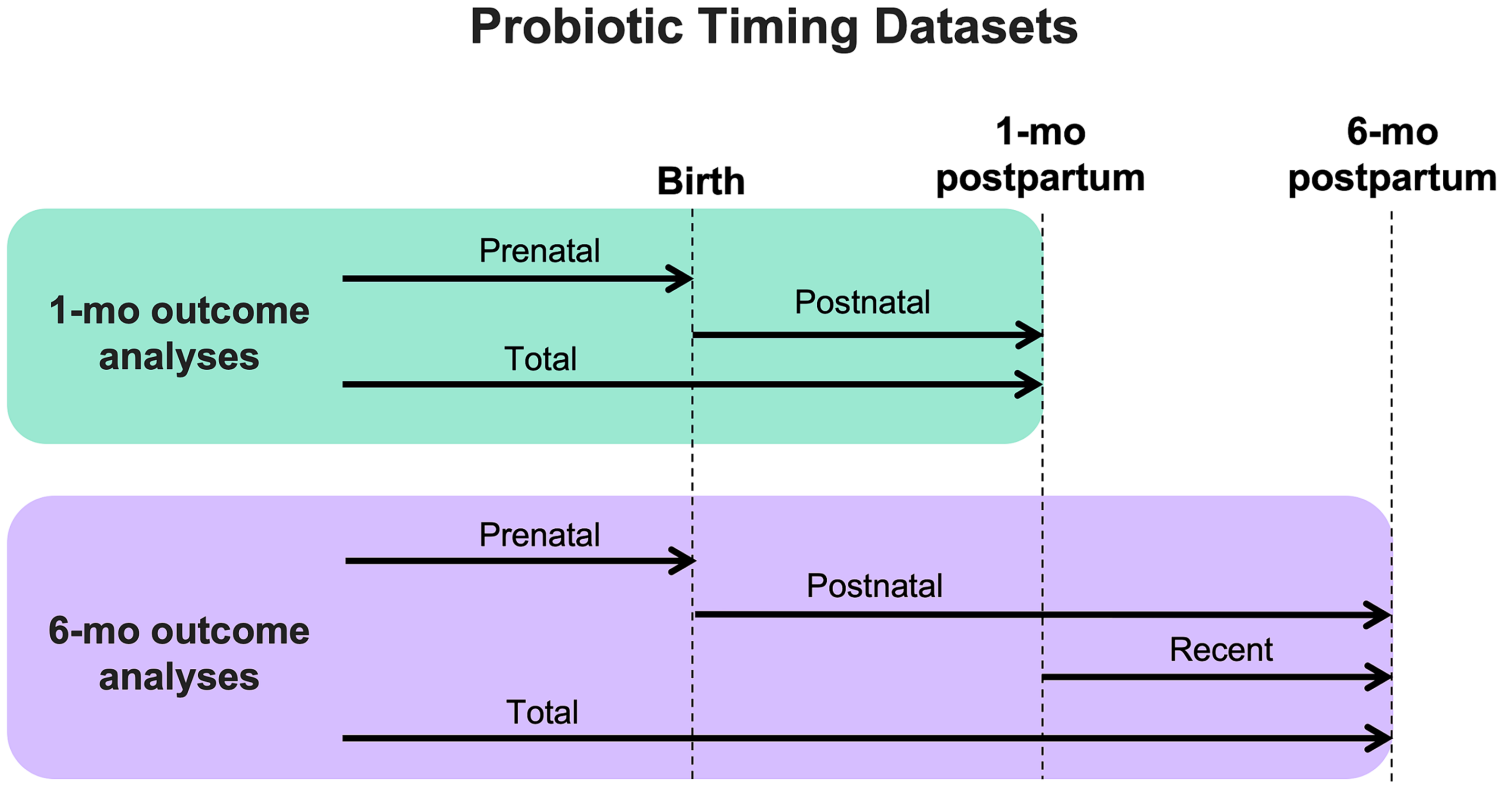
Diagram showing how outcome datasets were organized for analysis based on timing of maternal probiotic exposure.

Data regarding maternal probiotic exposure was collected via questionnaires administered during pregnancy and lactation (at 1-month and 6-month study visits) and included: use of probiotics (NO/YES), timing of probiotic use (before birth, between birth and 1-month study visit, and between 1- and 6-month study visits), and name of probiotic supplement (if known). Questionnaires did not specifically ask about yogurt consumption in the diet. In preliminary analyses, mothers and infants with less than 1 month of maternal probiotic exposure (5% of the cohort) had the same outcomes as those with NO maternal probiotic exposure (data not shown), and thus were included in the NO group. For all fecal and breastmilk analyses, probiotic groups (NO/YES) were further categorized by timing of exposure: prenatal, postnatal, and total (prenatal + postnatal) (Figure 1). For 6-month sample analyses, an additional exposure group was defined as those exposed to maternal probiotics after 1 month of age, to explore the association of more recent probiotic exposure on 6-month outcomes.

Due to the number of different probiotic supplements that the women in this cohort were exposed to (28 total), with only a small number of participants reporting use of any one of them, we did not assess differences in outcomes by specific probiotic supplement. A breakdown of known components in the probiotic supplements reported by subjects is detailed in Supplementary Table 2.1.

### 2.4 Sample collection and storage

Breastmilk and fecal sample collection and storage were performed as previously described (47). Briefly, breastmilk samples were obtained at a study visit when infants were 1 month old using a hospital-grade electric breast pump (Medela Symphony; Medela, Inc., Zug, Switzerland) and stored at −80°C within 20 min of collection. Fecal samples were collected at 1 and 6 months of infant age. Samples were either collected at a study visit, when feces were produced on site, or via a home collection kit. Parents and study staff were instructed to collect a pea-sized amount of freshly formed feces from diapers and place in a vial containing ethanol. Samples were transported or mailed to the study laboratory and then stored at −80°C upon arrival.

### 2.5 Inflammatory marker determinations

C-reactive protein (CRP) and interleukin-6 (IL-6) were quantified in breastmilk as described previously (51). Briefly, breastmilk was thawed, 1 mL of milk was centrifuged, and the fat layer was removed. Skimmed milk was then used with commercially available immunoassay kits to detect and quantify CRP and IL-6 (Abcam, Cambridge, MA; and R&D Systems, Minneapolis, MN; kits, respectively).

### 2.6 DNA isolation and microbial sequencing

DNA isolation from breastmilk and feces was performed as previously described (47). Briefly, DNA was extracted from 1 mL of skimmed breastmilk with the PowerSoil Pro kit (QIAGEN, Germantown, MD) and was stored in 100 μL of the kit elution solution at −80°C. DNA was extracted from thawed fecal samples using the PowerSoil kit (QIAGEN, Germantown, MD) and stored in 100 μL of kit elution solution at −80°C as previously described (47).

16S bacterial rDNA (V4 region) amplicons, generated by PCR, were sequenced by the University of Minnesota Genomics Center (UMGC, Minneapolis, MN) as previously described (52). Sequencing was carried out on an Illumina MiSeq system (Illumina, San Diego, CA) using V2 2×250 bp paired end chemistry. Raw sequences that passed quality control procedures were processed with the Shi7 (53) learning program, were aligned to reference databases using BURST (54, 55) generated from the 16S RefSeq collection compiled by the National Center for Biotechnology Information (NCBI), and taxonomy (genus level or higher) and reference tables were imported into RStudio.

### 2.7 Assessment of recognition memory function by event-related potentials (ERPs)

ERP measures of infant recognition memory performance were obtained at 1 and 6 months of age during follow up visits as previously described (56, 57). Briefly, a Hydrocel Geodesic sensor Net (Electrical Geodesics Incorporated (EGI), Eugene, OR) with 64 electrodes was placed on the infant’s scalp. For this study, we focused on ERP feature extractions from 4 scalp regions (left frontal, left central, right frontal, right central) that included 3 adjacent lead sensors each. These lead sensors were chosen based on beneficial characteristics for analysis: increased activity with least background noise for recognition memory task assessment, and with electrical activity at these leads expected to reflect synchronous activities of adjacent populations of neurons. At 1 month of age, infants were presented with a 750 msec recording of their mother’s voice or a stranger’s voice pronouncing the word “baby” alternating with equal but random probability. Voice recordings were digitized and edited using Creative Wave Studio; recordings were presented at 75.0 dB sound pressure. A total of 100 trials (50 of each voice) were presented. At 6 months of age, infants were presented randomly, one at a time for 500 msec each, with pictures of their mother’s face and stranger’s face, for a total of 100 trials (50 of each face). Pictures are standardized with respect to race, hair color, face and total picture size, neutral facial expression, and background color. Testing occurred in an electrically shielded room, and lights in the room were dimmed during stimulus presentation. EEG data were collected and recorded on-line using NetAmps Amplifiers (EGI, Inc., Eugene, OR) and NetStation software and referenced to a single vertex electrode (gain = 10,000x; filter = 0.1–100 Hz bandpass; sampling rate = 250 Hz).

Using NetStation analysis software (EGI), EEG data were filtered offline using a 30-Hz low-pass filter. Filtered EEG data from 1-month-old infants were divided into 2,100 msec segments (100 msec pre-event baseline, 750 msec stimulus presentation, and 1,250 msec post-stimulus onset, and baseline corrected to the average pre-stimulus voltage. Filtered EEG data from 6-month-old infants were divided into 1,600 msec segments) 100 msec baseline, 500 msec stimulus presentation, 1,000 msec post-stimulus onset. Data were visually inspected and excluded if they contained electrocardiographic or motion artifact. Consistent with previous research (58), trials were rejected if they contained more than nine artifact-contaminated channels, and these individual rejected channels on remaining trials were replaced using spherical spline interpolation. Subjects that contributed a minimum of 15 acceptable trials per condition were included in analyses; individual subject averages were calculated for each condition (mother/stranger) and re-referenced to the average reference.

Components of the electrical response reflecting hippocampus-based memory recognition and updating were the focus of this analysis. At 1 month of age, components of interest included P2 amplitude (adaptive mean within 200-500 msec window), an early sensory component modulated by memory, and the slow wave (SW, measured as mean amplitude of 900–2000 msec window). Following a familiar stimulus, the typical response is a return of the waveform to baseline after the P2 peak, reflecting that no further processing is needed. A negative slow wave after the P2 peak is seen in response to “new” stimuli and demonstrates novelty detection. Further, the slow wave difference score (familiar minus novel mean area amplitude from 900 to 2000 ms post stimulus) indexes discrimination between familiar and novel stimuli (38, 56, 59). Components of interest at 6 months of age included the negative component deflection (NC, adaptive mean within 350–700 msec window) which represents attentional response mediated by memory, and the SW (mean amplitude of 900–1,500 msec window). Like the 1-month paradigm, the SW reflects cognitive processing of the stimulus. At 6 months of age, we anticipate that infants would have fully encoded their mothers’ face, giving rise to a return to baseline after the NC while the stranger’s face would be partially encoded into memory (due to presentation in 50% of trials), evoking a positive slow wave, indexing memory updating (60). We also calculated a slow wave difference score (familiar minus novel SW mean amplitude from 900 to 1,500 msec) to indicate discrimination between the two stimuli.

### 2.8 Statistical analyses and data visualization

Welch’s t-tests were used to compare inflammatory marker levels between probiotic groups, with effect size (Cohen’s d) and 95% confidence intervals reported. RStudio (1.1.463) was used for microbiome feature extraction and to analyze microbiome data. Data analysis and visualization libraries used included: vegan, ape, nlme, ggplot2, ggbeeswarm, tidyverse, textshape, reshape2, randomcoloR, limma, ggsignif, Rtsne, igraph, gtools, and BiodiversityR. Euclidean dissimilarity beta-diversity measurements, using centered log ratio (CLR) normalized taxonomic counts (Aitchison’s distance), were compared using permutational analysis of variance (PERMANOVA) tests (Ashbury, et al.). *Lactobacillus* and *Bifidobacteria* abundances were compared between probiotic groups using a linear model with *p*-values, beta-coefficients, and 95% confidence intervals determined. Infant ERP features were analyzed only in the cohort of infants that provided fecal microbiome data at the corresponding time point. ERP features were compared in probiotic groups using linear models with *p*-values, beta-coefficients, and 95% confidence intervals reported. Clinical and demographic covariables were compared between probiotic (NO/YES) groups, for each probiotic timing category and by specific outcome, using Welch’s t-test (for comparison of HEI, a continuous variable) and chi-square test (for all other comparisons, categorical values) (Supplementary File 2; Supplementary Tables 2, 3). For all outcome analyses, covariables that differed between comparison groups were included in linear statistical models as indicated in the Results section. For all analyses, *p*-values <0.05 were considered statistically significant.

## 3 Results

### 3.1 Cohort description and comparison of clinical and demographic variables in probiotic groups and timing categories

The majority of women in the study cohort delivered infants vaginally (~83%). Pre-pregnancy BMI in the normal weight range was observed in ~38% of women, and the mean HEI was ~66, which is above the average reported for women by the U.S. National Center for Health Statistics (2017–2018). Similar numbers of women had optimal versus excess gestational weight gain (~48% vs. ~52%, respectively). Infant sex was balanced (~48% males vs. ~52% females) in the total cohort. White race was reported by mothers for ~80% of women and infants in the study cohort. With respect to antibiotic exposure, ~23% of women reported exposure to prenatal antibiotics. Postnatal antibiotic exposure between birth and the 1-month study visit was reported by ~48% of mothers, primarily related to antibiotics given at the time of birth for Cesarean section or positive screening for Group B Streptococcus. Exposure to antibiotics between the 1- and 6-month study visit was reported by ~14% of women. For infant postnatal antibiotic exposure, ~2% of infants received postnatal antibiotics prior to the 1-month study visit, and 15% prior to the 6-month study visits. Infant (as opposed to maternal) probiotic exposure was reported for ~2% of infants at 1 month and ~ 4% of infants at 6 months of age.

Maternal probiotic exposure (NO/YES) groups described 79 and 21% of women-infant dyads, respectively, in this study. Statistical comparisons of demographic and clinical variables were performed between the two probiotic groups (NO vs. YES), for each analysis type and time point and are shown in Supplementary Table 2.2 (1-month timepoint) and Supplementary Table 2.3 (6-month timepoint). This was done because not every subject provided data for all outcomes analyzed. Overall, the vast majority of covariates did not differ between probiotic groups. The few covariates that did differ were included in adjusted statistical models to understand their contribution to any significant differences in outcomes between probiotic groups, as detailed in the Results sections below.

### 3.2 Maternal probiotic exposure corresponds to lower levels of inflammatory markers in breastmilk

**Figure 2:**
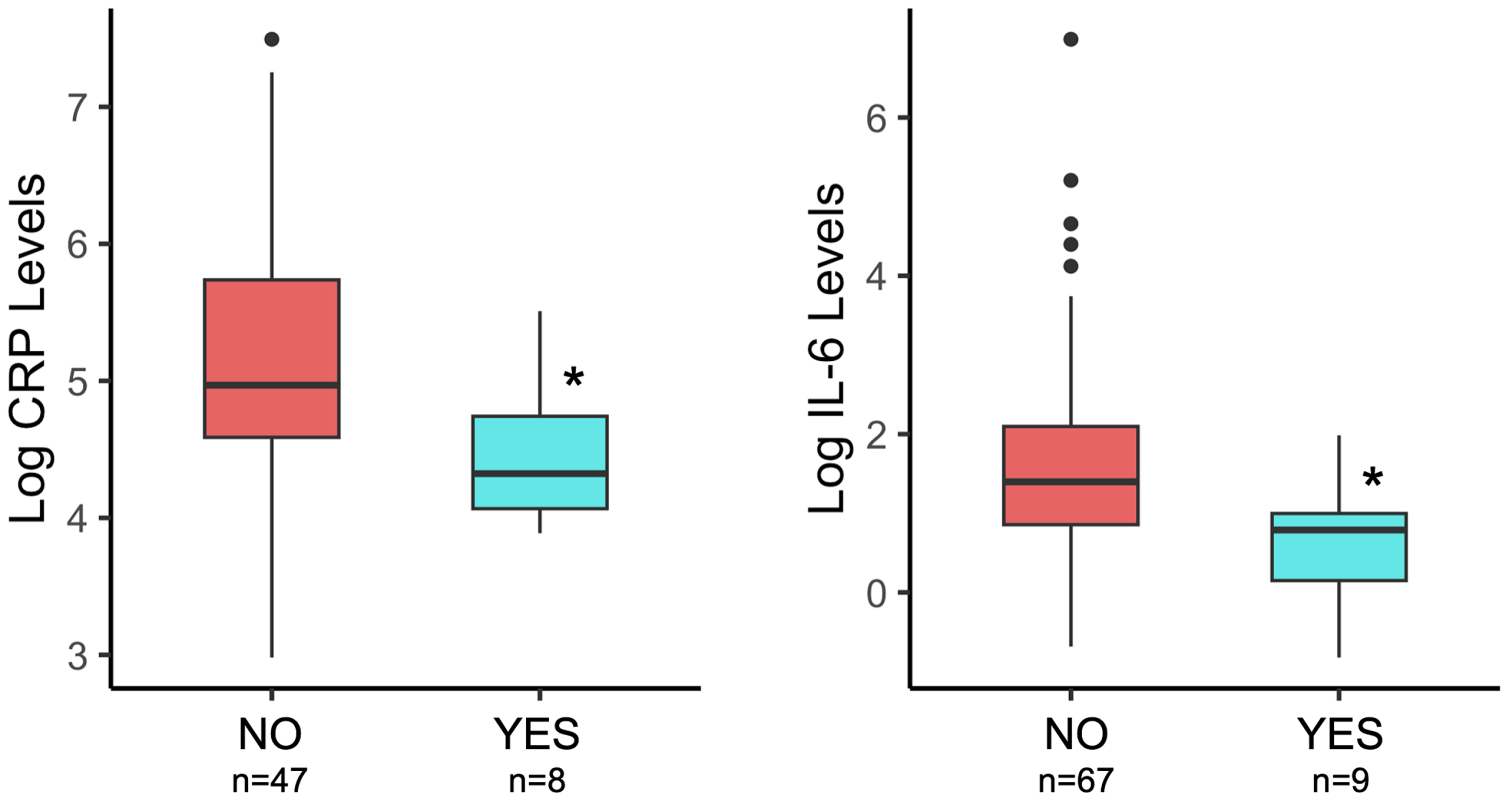
Comparison of breastmilk CRP and IL-6 levels by maternal probiotic group (NO/YES) and considering the total time of potential exposure (pre- plus post-natal). Details of statistical analyses are included in the Table 1. *p = 0.02 (CRP) and *p = 0.01 (IL-6).

Elevated levels of bioactive markers of inflammation have been implicated in mechanisms of how microbes contribute to negative health outcomes. Thus, we measured CRP and IL-6 levels in breastmilk, collected at 1 month postpartum, to understand how probiotic exposure is associated with milk inflammatory markers. Probiotic exposure (total) was associated with lower breastmilk C-reactive protein and IL-6 levels (Figure 2; Table 1) as compared to NO probiotic exposure. To explore how timing of probiotic exposure may be associated with inflammatory marker levels, subgroup analyses were performed for pre- and postnatal timing categories. Both pre- and postnatal maternal probiotic use were significantly associated with lower milk CRP concentrations. Differences in milk IL-6 levels were not observed for probiotic timing sub-categories (Table 1).

**Table 1:** Associations between maternal probiotic exposure at different timepoints (prenatal, postnatal, recent, and total) and infant fecal microbiome features, breastmilk inflammatory protein levels, and infant neurocognitive features at 1 and 6 months of age^1^

Outcome variable 1-month	Probiotic exposure timing		
	Prenatal	Postnatal	Total
	*p* value	*p* value	*p* value
Microbiome beta-diversity			
1 month infant fecal	**0.02** (*n* = 126, 16^2^)	0.46 (*n* = 132, 10)	**0.01** (*n* = 125, 17)
1 month breastmilk	0.34 (*n* = 100, 10)	0.28 (*n* = 103, 7)	0.37 (*n* = 98, 11)
*Lactobacillus* abundance			
Infant fecal	**0.001**, (*n* = 126, 16) β 0.85, CI [0.35, 1.36]	0.03^3^, (*n* = 132, 10) β 0.71, CI [0.07, 1.35]	**<0.001**, (*n* = 125, 17) β 0.96, CI [0.48, 1.45]
Breastmilk	0.20 (*n* = 100, 10) β 0.43, CI [−0.23, 1.08]	0.31 (*n* = 103, 7) β 0.40, CI [−0.37, 1.17]	0.32 (*n* = 98, 11) β 0.32, CI [−0.31, 0.95]
*Bifidobacteria* abundance			
Infant fecal	**0.01**, (*n* = 126, 16) β 0.66, CI [0.14, 1.17]	0.135 (*n* = 132, 10) β 0.49, CI [−0.15, 1.14]	**0.01**, (*n* = 125, 17) β 0.68, CI [0.18, 1.18]
Breastmilk	0.88 (*n* = 100, 10) β −0.05, CI [−0.71, 0.61]	0.74 (*n* = 103, 7) β −0.13, CI [−0.91, 0.65]	0.75 (*n* = 103, 7) β −0.10, CI [−0.73, 0.53]
Breastmilk CRP (log ng/ml)	**<0.05**, (*n* = 48, 7) d^4^ 0.75, CI [0.02, 1.46]	**0.03**, (*n* = 50, 5) d 1.02, CI [0.12, 1.88]	**0.02**, (*n* = 47, 8) d 0.78, CI [0.11, 1.44]
Breastmilk IL-6 (log ng/ml)	0.02^5^, (*n* = 69, 8) d 0.86, CI [0.15, 1.55]	0.08 (*n* = 72, 5) d 0.75, CI [−0.07, 1.53]	**0.01**, (*n* = 67, 9) d 0.91, CI [0.25, 1.55]

Outcome variable 6-month	Probiotic exposure timing			
	Prenatal	Postnatal	Recent	Total
	*p* value	*p* value	*p* value	*p* value
Microbiome beta-diversity (infant fecal)	0.66 (*n* = 105, 13)	0.16 (*n* = 95, 23)	**0.03**, (*n* = 98, 20)	0.12 (*n* = 92, 26)
*Lactobacillus* abundance (infant fecal)	0.89 (*n* = 105, 13) β −0.04, CI [−0.63, 0.54]	0.81 (*n* = 95, 23) β 0.06, CI [−0.41, 0.52]	0.50 (*n* = 98, 20) β 0.17, CI [−0.32, 0.65]	0.496 (*n* = 92, 26) β 0.15, CI [−0.29, 0.59]
*Bifidobacteria* abundance (infant fecal)	0.08 (*n* = 105, 13) β 0.52, CI [−0.06, 1.09]	**0.03**, (*n* = 95, 23) β 0.51, CI [0.06, 0.96]	0.07 (*n* = 98, 20) β 0.44, CI [−0.04, 0.93]	**0.02**, (*n* = 92, 26) β 0.51, CI [0.08, 0.95]
ERP, slow wave difference score, by EEG lead (μv)				
Left central	**<0.05**, (*n* = 49, 5) β 0.93, CI [0.02, 1.85]	**0.04**, (*n* = 44, 10) β 0.71, CI [0.03, 1.39]	**0.01**, (*n* = 47, 7) β 1.02, CI [0.25, 1.79]	**0.04**, (*n* = 44, 10) β 0.71, CI [0.03, 1.39]
Left frontal	0.96 (*n* = 49, 5) β 0.03, CI [−0.93, 0.98]	0.10 (*n* = 44, 10) β 0.58, CI [−0.11, 1.27]	**0.03**, (*n* = 47, 7) β 0.89, CI [0.11, 1.68]	0.10 (*n* = 44, 10) β 0.58, CI [−0.11, 1.27]
Right central	0.49 (*n* = 49, 5) β −0.33, CI [−1.28, 0.62]	0.46 (*n* = 44, 10) β −0.26, CI [−0.97, 0.45]	0.38 (n = 47, 7) β −0.36, CI [−1.17, 0.46]	0.46 (*n* = 44, 10) β −0.26, CI [−0.97, 0.45]
Right frontal	0.73 (*n* = 49, 5) β 0.16, CI [−0.79, 1.11]	0.06 (*n* = 44, 10) β 0.65, CI [−0.04, 1.33]	**0.01**, (*n* = 47, 7) β 1.08, CI [0.32, 1.85]	0.06 (*n* = 44, 10) β 0.65, CI [−0.04, 1.33]

^1^Statistical comparison as noted in Methods for each outcome, by probiotic group (NO vs. YES) with significant differences noted in bold.

^2^*n* by probiotic group (NO, YES).

^3^Not significant when infant probiotic exposure was added to statistical model.

^4^Cohen’s d.

^5^Not significant when maternal age was added to statistical model.

### 3.3 Maternal probiotic exposure is associated with variation in infant fecal, but not breastmilk, microbiome composition

**Figure 3:**
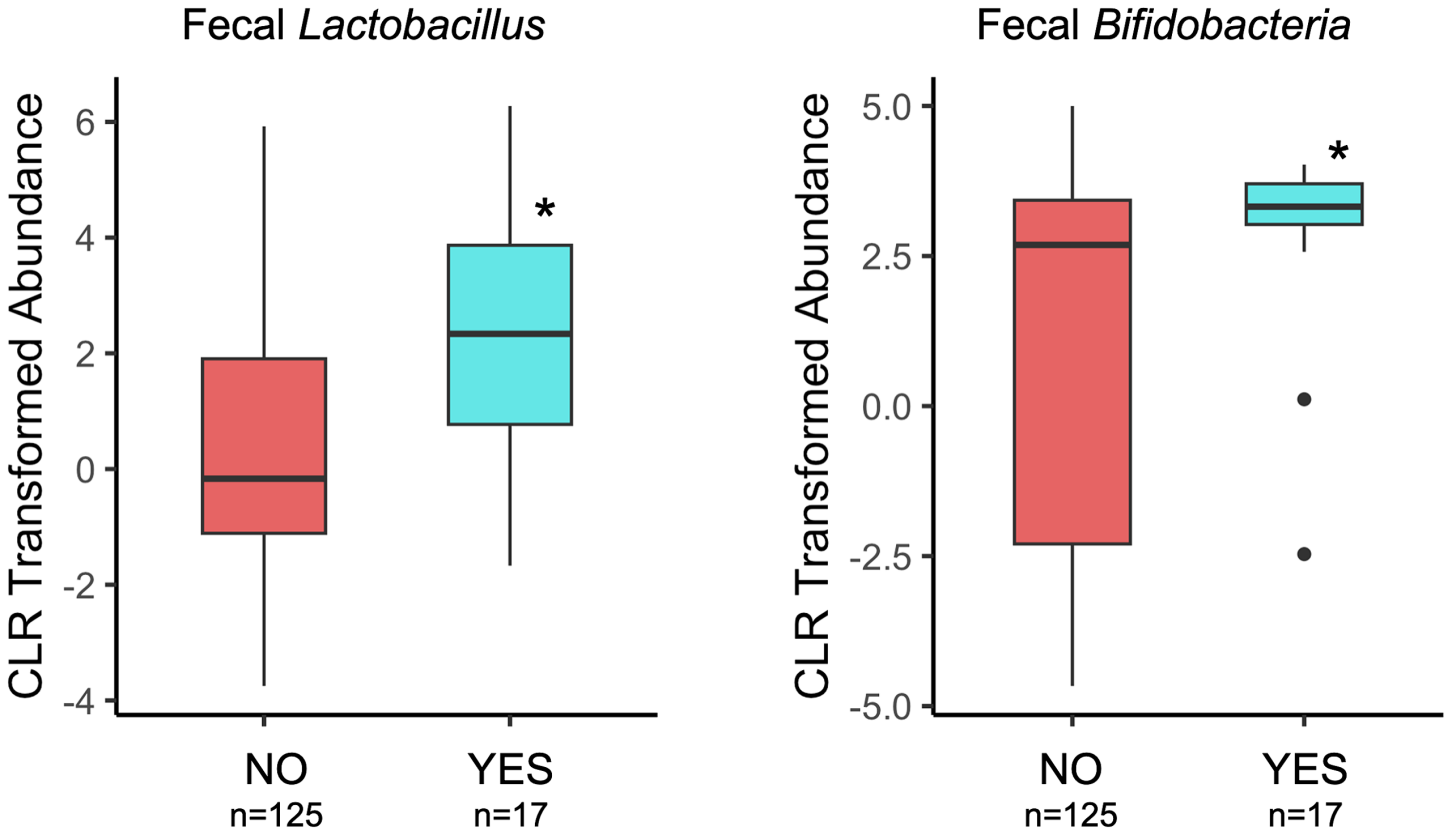
Comparison of *Lactobacillus* and *Bifidobacteria* relative abundances in 1-month infant feces by maternal probiotic group and considering the total time of potential exposure (pre- and post-natal). Details of statistical analyses are included in the Table 1. *p < 0.001 (genus *Lactobacillus*) and *p = 0.01 (genus *Bifidobacteria*).

Infant fecal microbiome composition (beta-diversity) at 1 month of age significantly differed between probiotic exposure (NO/YES) groups (Table 1) when considering total time (prenatal and postnatal) of exposure. In subgroup analyses, prenatal, and not postnatal, exposure was significantly associated with infant fecal microbiome variation (Table 1). Maternal age was higher in the probiotic “YES” groups for the prenatal and total timing categories at 1-month Supplementary Table 2.2. When maternal age was included in the statistical models, we observed that probiotic groups remained different with respect to microbiome compositions, indicating that maternal age is likely not contributing to these associations. We also compared the fecal abundances of “beneficial” bacterial taxa, *Lactobacillus* and *Bifidobacteria*, between probiotic groups. Of note, *Lactobacillus* was a component in the majority of probiotic supplements that were self-reported by the women in this cohort (~94%, Supplementary Table 2.1). At the 1-month timepoint, the probiotic (YES) group of infants had significantly higher abundances of fecal *Lactobacillus* and *Bifidobacteria* in the prenatal and total timing categories (Figure 3; Table 1). These differences remained significant when maternal age, the only covariable that differed for these probiotic groups and timing categories, was included in the statistical models. In contrast to the results with infant feces, 1-month breastmilk microbiomes did not differ with respect to composition or differential abundances of taxa, based on probiotic exposure, for any probiotic timing category.

At 6 months of infant age, probiotic (NO/YES) groups also had different fecal microbiome compositions, but only for the subgroup that had postnatal maternal probiotic exposure after 1 month of age (Table 1). This may indicate that probiotic exposure closer to the time of sample collection is more important to fecal microbiome variation. The association of 6-month microbiome variation with more recent exposure of infants to maternal postnatal probiotics remained significant when the statistical model was adjusted for infant probiotics, the only covariable that differed between probiotic groups for this exposure time. With respect to taxa abundances, maternal postnatal and total probiotic exposure were associated with higher abundance of fecal *Bifidobacteria* in 6-month-olds. There were no differences in *Lactobacillus* abundances for any timing category (Table 1).

### 3.4 Recent postnatal maternal probiotic exposure was associated with variation in ERP responses in 6-month-old infants

**Figure 4:**
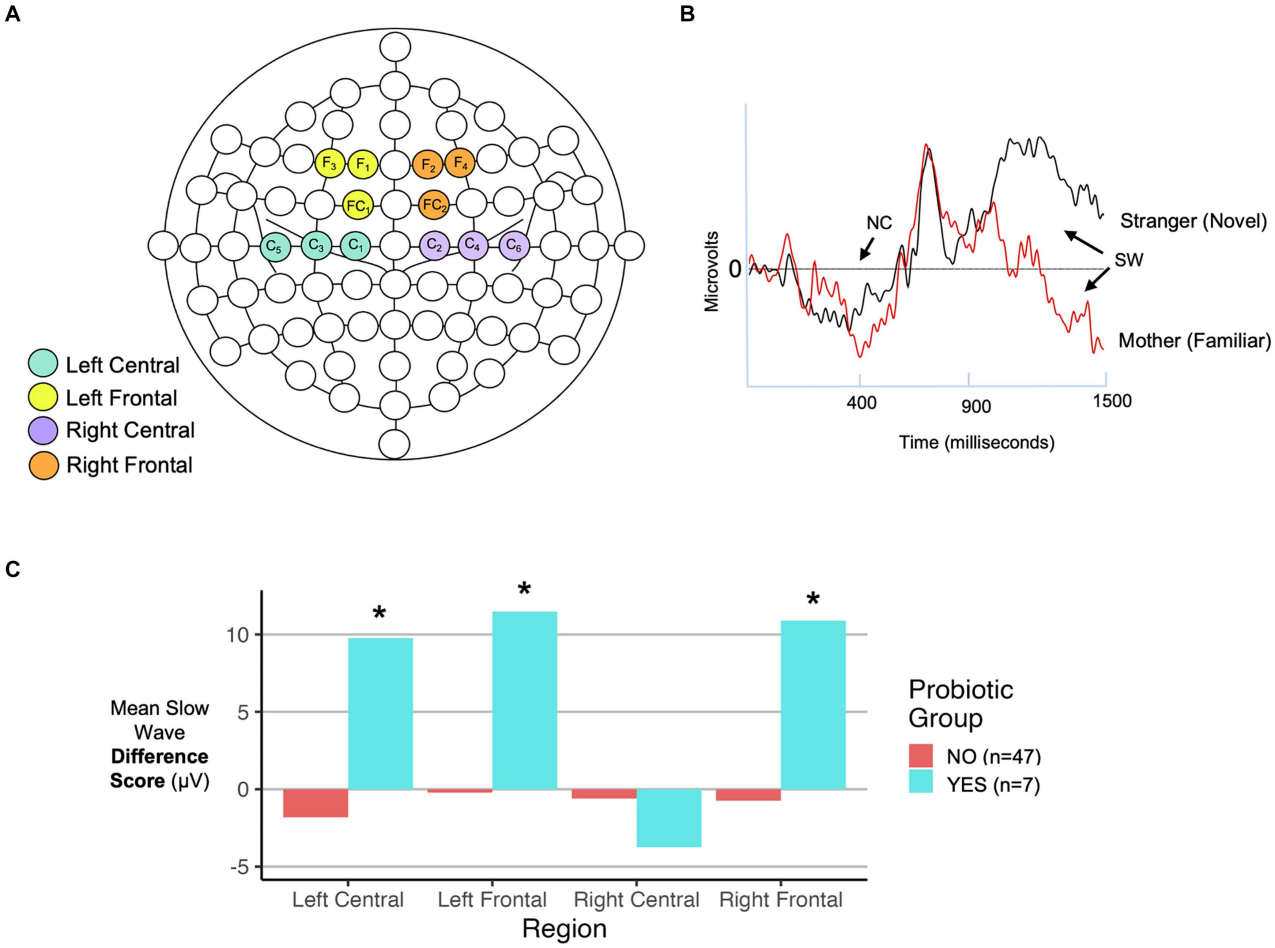
Infant ERP analysis features. **(A)** EEG net map with electrodes highlighted that correspond to scalp regions analyzed in this study. **(B)** Representative ERP recording with locations of the negative component (NC) peak at approximately 400 ms (350–700 ms time range analyzed) and slow wave (SW) from 900-1500 ms indicated with arrows. Two responses are shown: one to a familiar (own mother-based) and one to a novel (stranger-based) stimulus. **(C)** Comparison of SW difference scores (familiar - novel) by probiotic exposure group in 6-month-old infants for each of the four scalp regions indicated in panel **(A)**. Results are shown for infants exposed to recent postnatal maternal probiotics. Details of statistical analyses are included in the Table 1. *p < 0.05.

To compare infant recognition memory function in probiotic groups, ERP components of interest were extracted from infant EEGs that were obtained in response to auditory (1 month) or visual (6 month) stimuli as described in the Methods. We observed larger slow wave difference scores in 3 of 4 scalp EEG regions, for 6-month-old infants exposed to more recent maternal probiotics (after 1 month of age) (Figure 4; Table 1), potentially indicating a greater stimulus discrimination. This subgroup of infants with larger difference scores and more recent exposure to maternal probiotics also had different 6-month fecal microbiome compositions (PERMANOVA, *p* = 0.02, *n* = 39 NO probiotic and *n* = 7 YES probiotic) and lower breastmilk IL-6 levels (Welch’s t-test, *p* = 0.004, Cohen’s d 1.14, CI [0.35, 1.90], *n* = 39 NO probiotic and *n* = 7 YES probiotic). In this subgroup, *Lactobacillus* and *Bifidobacteria* abundances did not differ in 6-month feces between probiotic NO/YES groups. We were unable to test for associations with other outcomes (milk CRP and 1-month fecal microbiome features) in this subgroup due to a lack of adequate subject numbers in the probiotics YES group to enable a statistical comparison. We also observed ERP differences (in late slow wave difference scores) at 6 months of age for the prenatal probiotic timing category, but only in the left central scalp lead (Table 1). For 1 month ERP testing, we did not find any significant associations between probiotic exposure (any timing group) and differences in ERP features in any scalp electrode region.

## 4 Discussion

Consistent with the Developmental Origins of Health and Disease Hypothesis, nutritional factors are one type of environmental exposure during early life that have the potential to affect long-term health outcomes. Establishment of gut microbial communities during infancy is largely shaped by diet, and probiotics are thought to promote healthy microbiomes that confer health benefits. The overarching objective of our research is to explore how microbes, a type of nutritional outcome/factor, are linked to the development of brain function during early human life. In this pilot observational study, we aimed to characterize how maternal dietary supplementation with probiotics is associated with several features that have been proposed to be important for the function of the Microbiome-Gut-Brain Axis (1, 31, 33) namely inflammation (via assay of breastmilk inflammation-related proteins), the gut microbiome of infants, and infant brain function. To enhance the potential, for hypothesis generation for future studies, we maximized comparison group sizes by initially analyzing for associations between maternal probiotic exposure and each outcome individually. We found associations between maternal probiotic use and (1) decreased breastmilk CRP and IL-6 levels, (2) differences in infant fecal microbiomes at 1- and 6- months of age, and (3) differences in ERP responses for a subgroup of infants with more recent exposure to maternal probiotics. In an exploratory subgroup analysis of these infants with larger ERP difference scores at 6 months of age and a complete data set for all outcomes, maternal probiotic exposure was also associated with decreased IL-6 levels as well as variation in 6-month fecal microbiome composition. These findings suggest that probiotic usage in pregnant and lactating mothers may alter the Gut-Microbiome-Brain Axis in human infants.

Probiotic supplementation during pregnancy and lactation has been associated with a reduction in maternal inflammation-associated proteins. Pregnant women who consumed probiotic yogurt had decreased levels of CRP in their blood (27, 28), and decreased levels of breastmilk IL-6 were reported in women using probiotics during late pregnancy and lactation (61). We also found decreased CRP and IL-6 levels in the breastmilk of mothers exposed to probiotics, consistent with these prior reports. The mechanism by which maternal probiotics influence breastmilk inflammation-associated proteins has not been clearly defined. One potential hypothesis is that probiotic organisms, or altered microbiomes resulting from probiotic organisms, modulate inflammatory responses in the maternal gut which are then transferred from the gut to the systemic circulation and then to the breastmilk. Alternatively, probiotic-altered maternal gut microbiomes could be transferred to the breast, as has been proposed in the enteromammary circulation mechanism (62), to affect inflammation responses directly at that site. Although we did not find modulation of breastmilk microbiomes to be associated with probiotic exposure, as has been reported by other studies (63), this does not eliminate the possibility that changes to milk microbes and/or their functions (not able to be detected by our methods) are playing a role in the regulation of milk cytokine levels by maternal probiotics.

Maternal probiotic supplementation was also associated with variation in the composition of infant gut microbiomes at both 1 and 6 months of age in our study cohort, consistent with previous reports. Of note, *Lactobacillus* and *Bifidobacteria* were more highly abundant in 1-month fecal microbiomes of infants exposed to maternal probiotics, and *Bifidobacteria* was more abundant in 6-month-old infants exposed to postnatal maternal probiotics. *Lactobacillus* and *Bifidobacteria*, components of many of the probiotic supplements used by the women in our cohort, are beneficial bacteria that have been associated with decreased gut inflammation and improved health outcomes in infants, including atopic dermatitis and food allergy (64), necrotizing enterocolitis (65) and infantile colic (66). Consistent with our results, a recent meta-analysis reported that probiotic supplementation of mothers during pregnancy and lactation is associated with increased abundances of the probiotic bacteria in their infants’ feces, with abundances peaking near the first month of life (67). Although it appears that probiotic organisms are not sustained long-term in the infant gut, it remains unclear if early, transient colonization could be sufficient to modulate long-term outcomes.

The hippocampus, a brain region involved in recognition learning and memory, has been shown in rodent studies to be a primary brain structure affected by gut microbes (1, 35, 36). In addition, a recent study (68) reported that species within the *Bifidobacterium* genus isolated from infant feces along with specific human milk oligosaccharides from their mothers’ milk, were associated with early cognitive development, highlighting the idea that specific microbes may play a role in neurodevelopment during critical periods (69–71). Based on these prior studies, we hypothesized that ERP measures of recognition memory may differ based on exposure to maternal probiotics in human infants. We observed that in 6-month-old infants who were exposed to more recent maternal probiotics, the difference in ERP response (slow wave mean area amplitude) to familiar and novel stimuli was significantly larger for the majority of scalp regions measured. The larger difference score in these infants may indicate a greater ability to discriminate between familiar and novel stimuli in the probiotic-exposed group, or, in other words, enhanced recognition memory function (56, 59). This altered recognition memory could be due to structural or functional changes in hippocampus-based brain circuits associated with the Microbiome-Gut-Brain axis. The exact processes underlying how gut microbes affect brain function are not yet completely understood, but proposed mechanisms in animals include modulation of expression of host and/or microbial genes and proteins involved in neurotransmission, synaptic plasticity [e.g., Brain Derived Neurotrophic Factor (BDNF)], metabolism (e.g., short-chain fatty acids), stress hormones, gut-brain neuronal signaling via the vagus nerve, and brain structure (1, 2, 36, 72).

This pilot study has limitations. First, because of the observational study design, our results could be affected by a lack of consistency in the type and duration of probiotic exposure as well as the relative low number of infants exposed to maternal probiotics as compared to those who were not. Follow-on controlled clinical trial studies are needed to validate and expand on the findings of our study. Second, the lower subject numbers from which we had a complete set of outcome data limited our ability to characterize complex interactions among all outcomes. Third, complete sample and outcome collection at more frequent time points and over a longer period of time (e.g., first 2 years of life) would have afforded the opportunity to better identify time-dependent outcome associations with probiotic exposure. Our study also has key strengths. First, we leveraged a unique cohort of exclusively breastfeeding mother-infant dyads; thus, infant results were not confounded by variation in their diet (addition of formula or solid foods). Second, we used ERPs to assess brain (recognition memory) function in infants. ERP can be performed in much younger (preverbal) infants than traditional cognitive testing; assessment closer to the time of birth has the potential to identify relationships between exposures (relevant specifically to the breastfeeding period) and brain function with minimum confounding or dilution of effects by subsequent exposures. ERP responses have been validated in small cohort sizes to be able to detect important differences between groups (38, 59, 73). Overall, we argue that this study adds to the growing literature regarding maternal probiotic use to optimize infant outcomes. These preliminary data are important in that they have provided additional hypotheses that can be used to design future studies aiming to develop early probiotic supplementation strategies to optimize infant neurodevelopmental outcomes.

## Data Availability

Sequence and code data sets presented in this study can be found in online repositories. Raw DNA sequence data sets for this study are available in BioProject PRJNA880162 at NCBI (https://www.ncbi.nlm.nih.gov/bioproject/PRJNA880162/). Computer code for statistical models and experimental data tables are available at: https://github.com/CGaleLab/Probiotic_Microbiome_Infant_Neurodevelopment.
